# Exploring Lived Experiences of Vulnerability in Nursing Management during the Coronavirus Disease 2019 Pandemic: A Phenomenological Study of Nurse Managers and Nurses

**DOI:** 10.1177/23779608241286308

**Published:** 2024-10-08

**Authors:** Nastaran Heydarikhayat, Nezar Ghanbarzehi, Fatemeh Darban, Zahra Atarodi Kashani, Camelia Rohani

**Affiliations:** 1 Department of Nursing, School of Medicine, 461251 Iranshahr University of Medical Sciences, Iranshahr, Iran; 2 Department of Midwifery, School of Medicine, 461251 Iranshahr University of Medical Sciences, Iranshahr, Iran; 3 Department of Health Care Sciences, Palliative Care Research Center, Marie Cederschiöld Högskola, Stockholm, Sweden; 4 Department of Community Health Nursing, School of Nursing and Midwifery, Shahid Beheshti University of Medical Sciences, Tehran, Iran

**Keywords:** vulnerability, healthcare organization, crisis management, nurse, COVID-19

## Abstract

**Introduction:**

The Coronavirus Disease 2019 (COVID-19) pandemic placed enormous pressure on healthcare systems around the world, but it also provided valuable insights for healthcare organizations during this challenging period.

**Objective:**

This study aimed to explore nurses’ lived experiences of vulnerability in hospital nursing management during the COVID-19 pandemic, and reflect on the lessons learned.

**Methods:**

In this phenomenological study, 14 participants, including nurse managers at different levels and staff nurses, were selected by purposive sampling from one center university hospital. Data collection was done through in-depth individual semistructured interviews with participants and a review of weekly reports of crisis management meetings at the university hospital. Interviews were analyzed using Colaizzi's method in seven phases by MAXQDA software Version 10.

**Results:**

One overarching theme, four themes, and 15 subthemes were obtained from analyses of interviews. Four themes of “nurses’ attrition,” “distrust of society to the organization,” “fragility in the organization's performance,” and “intensified inequalities” were extracted as threats to nursing management at the hospital. Subsequently, the weekly reports of crisis management meetings at the university hospital were analyzed to extract the solutions and lessons.

**Conclusion:**

The unpreparedness of the healthcare system against a crisis can led to the loss of organizational assets, including medical staff and the credibility of the healthcare system. Limitations of the infrastructure at hospital became more obvious during the pandemic and caused serious threats to the healthcare system. Despite severe challenges along with the pandemic, it offered four valuable lessons in nursing management.

## Introduction

Comprehensive crisis management is an important and controversial challenge in the field of management (Permatasari & Mahynui, 2022). Among different components of crisis management, healthcare systems play an essential role in management and control of the crisis. Healthcare systems are responsible for presenting the plan preparations and strategies to deal with the crisis to reduce the loss of life. Many high-level crisis managers face numerous problems in practice (Arboleda et al., 2009; Labaf et al., 2021; Liu et al., 2020). One of these troubling challenges is vulnerability. Vulnerability is the degree of adverse response to a system or part of it when faced with a risk (Proag, 2014). Vulnerability refers to the sensitivity or weakness of a specific population or organization to the harmful effects of exposure to high-risk events. Severe consequences can result from vulnerability and unpreparedness for disasters. Furthermore, a threat has the potential to increase vulnerability (Du et al., 2015). We have all witnessed how the Coronavirus Disease 2019 (COVID-19) pandemic, exposed all healthcare systems to vulnerability (Siddique et al., 2021).

A realistic glance at the available evidence shows that the COVID-19 pandemic not only had a negative impact on the healthcare systems worldwide, but it also produced great lessons in the field of crisis management (Moyo et al., 2022). During this period, healthcare systems encountered many challenges due to the COVID-19 pandemic (Jabbari et al., 2022). The pandemic weakened even the strong healthcare systems around the world, and this led to failures in the management and appropriate response to the crisis. Inadequate resources were reported along with deficiencies in the infrastructure of healthcare systems (Filip et al., 2022). Challenges in healthcare systems not only included job stress and inconsistent policies, but also included shortages of resources and financial problems (Sengupta et al., 2021). The spread of COVID-19 put pressure on all different parts of healthcare systems and created a strong demand for nurses and other healthcare providers around the world.

Nursing, inherently, is a stressful profession and the spread of the COVID-19 pandemic also increased the levels of stress among nurses (Zamanzadeh et al., 2021). Nursing managers, as the first-line defenders in healthcare organizations, have an essential role in managing and coordinating. They have always been important organizers at hospitals during crisis situations. In addition to patient care, nurses have other responsibilities, such as managing healthcare facilities, mitigating vulnerability, threats, and risks, promoting productivity, and improving efficiency and sustainability (Moyo et al., 2022). The results of a qualitative study during the COVID-19 in Iran showed that despite the critical role of nurses in the initial part of the crisis, they did not have sufficient preparation nor enough facilities to deliver necessary healthcare services (Shah Talebi et al., 2021).

## Literature Review

A review of the literature shows that there were various challenges in hospitals during the spread of COVID-19. These challenges included the lack of effective management, inappropriate policy making, unsuitable planning, not having updated guidelines, shortage in the number of nursing staff and other healthcare providers, infection among nurses, no access to enough personal protective health equipment and necessary sanitary supplies, as well as a shortage of medicine and payment discrimination for delivering healthcare services (Cohen & van der Meulen Rodgers, 2020; Hossny et al., 2022; Jabbari et al., 2022; Kwon et al., 2022). Furthermore, the results of previous studies showed that caring for COVID-19 patients was a challenging experience for nurses (Kwon et al., 2022; Roe et al., 2022). Evidence shows that the U.S. nursing workforce has been under significant pressure due to high workloads and increased levels of burnout caused by the COVID-19 pandemic, with the impact more pronounced among younger and less experienced nurses. In addition, ineffective regulatory policies, poor policy making, and inadequate workforce planning, as well as not enough leadership management and social support, job dissatisfaction, difficult conditions at work, and changing working hours during caring for patients with COVID-19 and psychosomatic disorders, exacerbated the nursing labor shortage (Poortaghi et al., 2021; Tamata & Mohammadnezhad, 2023). Therefore, during the pandemic, healthcare managers tried to be more flexible and assess the situation before decision making (Poortaghi et al., 2021). Earlier studies indicated that dealing with an unknown disease, organizational problems such as shortages of facilities and getting along with healthcare structural limitations and risk of infection were important challenges during the COVID-19 pandemic (Darvishpour & Nikfam, 2023; Podgorica et al., 2022). Based on the results of a phenomenological study in the United States, nurse managers and their assistants experienced three types of leadership challenges during the period of COVID-19, that is, using a different kind of support for their personnel and focusing on emotional support, renewing their personal approach and managing resources and having continuous communication due to changes in nursing protocols, and also confronting nurses’ avoidance fear of providing care for COVID-19 patients (White, 2021). Moreover, Turkish nurse managers experienced several challenges, including changes in their roles, tensions at the workplace, and psychological and physical problems (Aydogdu, 2023).

Since, transparency in challenges and resolution of them at all levels of healthcare organizations help improve crisis management, we decided to conduct this phenomenological study to explore lived experiences of vulnerability in hospital nursing management among nurse managers and nurses during the spread of COVID-19 and reflect on the lessons learned.

## Research Question

**“**How was the vulnerability of hospital nursing management during the spread of COVID-19?”

## Methods

### Research Design

This study employed a qualitative research design with a phenomenological approach that was approved by the research ethics committee of the university (ethical code: IR.IRSHUMS.REC.1401.001). Phenomenology is a philosophy that focuses on understanding human experiences and how researchers can uncover and analyze the essence of these phenomena. It seeks meaning from reality through deep energumen (Al-Sheikh Hassan, 2023; Shosha, 2012). Edmund Husserl is recognized as the primary founder of descriptive phenomenology in the early twentieth century. The purpose of descriptive phenomenology is to describe the essence of the phenomenon under study as perceived by those who are directly involved with it (Sinfield et al., 2023; Skoglund et al., 2023). The essence of a phenomenon refers to a structure of basic meanings that clarifies a particular phenomenon. The essence defines the main characteristics of a phenomenon; without these features, the phenomenon loses its identity (Dahlberg, 2006). One of the fundamental aspects of Husserl's phenomenology is the concept of intentionality. This concept, which pertains to the manner in which consciousness is oriented toward an object or phenomenon, serves as a pivotal element in Husserl's philosophical framework. Husserl posits that all forms of consciousness are inherently directed “toward” something, indicating that our cognitive activities are perpetually focused on objects or events in the external environment as well as within our subjective experiences (Christensen et al., 2017; Mahfuz, 2024). In this study to understand the reality, intentionality was attained through observing, thinking about, immersing within the phenomenon, and focusing on the content (by asking “what is she/ he thinking?”).

## Study Setting

Data were collected from a number of nursing managers and nursing staff at a university hospital in southeast of Iran. It was the only center in the city which was assigned to daily admission of COVID-19 and non-COVID-19 patients. Four wards in this hospital were dedicated to the care of suspected or positive COVID-19 patients. During the COVID-19 pandemic, 220 nurses worked in the university hospital. Hospital is affiliated with a University of Medical Sciences which is located in the Sistan and Baluchestan province. This province has seven main cities with a population around 2,534,327 people in an area of 181,578 square kilometers (University of Sistan and Baluchestan, 2024).

### Participants and Data Collection

All nurse managers from one university hospital were invited to participate in the study (*n* = 24). Participants included 10 nursing managers at different levels (head nurses, clinical and educational supervisors, and director of nursing services) and four staff nurses who were working in COVID-19 and non-COVID-19 units (*n* = 14). They were entered into the study through purposive sampling to ensure they had the required experience about the phenomenon (Ahlqvist et al., 2023). Purposive sampling is a frequently utilized method in qualitative research to identify and choose participants that offer valuable information pertaining to the phenomenon of interest (Palinkas et al., 2015). It enhances the congruence between the sample and the study's objectives and leads to an increase in the rigor of the study (Campbell et al., 2020).

Inclusion criteria for participants were as follows: being a nurse manager and/or a staff nurse at the university hospital with at least 6 months experience in the same hospital during the COVID-19 crisis. They were selected with the greatest possible variability according to age, marital status, professional experience, being in three management levels, and activity as a staff nurse in the university hospital wards.

The present study was conducted from 19th of May to the end of August 2022. At the time of sampling, there was the seventh wave of COVID-19 in Sistan and Baluchestan. Based on the color-coded COVID-19 warning, the level of transmission of the disease in different parts of the city was yellow (relatively risk) and blue (minimal risk), while in other parts of the country, the number of red cities had increased (Ghotbi, 2022).

Data were collected through a series of individual, in-depth and semistructured interviews from 14 participants. The face-to-face interviews were conducted in Persian by two male and female experts in qualitative research, the authors NH (PhD) and NG (MSc), who were faculty members and mentor of nursing students in the same university hospital. The participants were recruited through formal contacts by phone or direct visit at the hospital. The interviewers accessed to the participants in different working shifts in the hospital. Due to the heavy workload of nursing managers, several visits were made to meet them in the hospital. Nurse managers at different levels and staff nurses were asked regarding their willingness to participate in the study. After a comprehensive explanation of the research objectives and obtaining oral and written consent, the preferred time and place of the interview were determined by the participants.

Fourteen interviews were conducted with nurse managers and staff nurses. The time of interviews was between 35 to 60 min depending on the participant's responses. An interview guide was used to conduct the interviews (Turner, 2010). It included several open-ended questions, and the rest of the questions were generated based on the participants’ responses. In the first two interviews, the sample questions of the interview guide were pilot tested. At the end of each interview, the participants were informed that they would receive a phone call or message (through WhatsApp) to ensure that their experience of the phenomenon under study is accurately represented in the findings. To start off the interviews, these questions were asked: “Please explain how did you experience vulnerability in nursing management during the COVID-19 pandemic?” “Based on your experience, which factors had exacerbated vulnerability in hospital nursing management?” The interview was then continued with probing questions, such as “Please explain more?” and “Could you give me an example?” All interviews were audio-recorded after obtaining permission by signing the Informed Consent Form from all participants. Data saturation occurred on the 12th interview, but for more assurance, they continued with two more interviews. Data saturation plays an important role in qualitative research as it determines when data collection and/or analysis should be stopped (Hennink & Kaiser, 2022). The adequacy of a sample for a study can be determined by saturation, which indicates whether the data gathered accurately reflects the diversity, complexity, and subtleties of the subjects being examined (Rahimi, 2024). The process of bracketing commenced at the initial stage when the project was conceptualized and persisted throughout the study. All the presuppositions and preconceptions that resulted from the researcher's experience were bracketed before the study. Bracketing was done by writing memos during data collection and data analysis. The rationale behind selecting the research topic along with the assumptions and preconceptions about management styles within the healthcare system and the managers’ age, their gender, the cultural context, and limited resources in the hospital were documented prior to the study. Additionally, the beliefs of the local population regarding herbal and traditional remedies, the belief in transferring patients to well-equipped medical facilities in neighboring provinces, and the researcher's personal values were also outlined in advance of the research. Bracketing has its roots in the phenomenology tradition. Researchers utilize bracketing as a technique to minimize the negative impacts of undisclosed preconceptions associated with the research, ultimately enhancing the study's rigor. It protects the researcher from the emotionally challenging materials and helps them to get deeper levels of reflection during the qualitative phases of the study (Tufford & Newman, 2012). Despite the fundamental philosophical distinctions between the terms epoché and bracketing, they are often used interchangeably. Epoché is defined as a continuous analytical process that must be actively integrated into the sequential development of the research from the beginning of the study. In contrast, bracketing occurs during the interpretive phase. Unlike bracketing, which is limited to a specific stage and episode before interpretation, the concept of epoché involves continuity and sequence (Bednall, 2006).

In the next step, a review was done on the weekly summary reports of crisis management meetings at the university hospital. This meeting consisted of a panel of experts with nurse managers, head nurses, and senior managers of the university. All issues and serious challenges were discussed and reviewed daily and/or on an emergency basis through an online meeting. The results of these meetings were recorded on the online crisis management platform of the hospital's website. This platform was accessible only by managers at the hospital and headquarters for dealing with COVID-19 in the university.

### Data Analysis

The data collected during the interview were analyzed either on the same day or the following day using Colaizzi's descriptive phenomenology. The data management was facilitated by MAXQDA software Version 10 (ver. 10 R 160410; Udo Kuckartz, Berlin, Germany). Descriptive phenomenology aims to uncover the essence or core structure of any phenomenon being studied. That is, it focuses on the distinctive features that create that phenomenon (Morrow et al., 2015). The Colaizzi's method consists of seven steps: familiarization by immersing researchers in data and a full understanding of participants’ experiences by transcribing interviews verbatim; identifying significant statements by extracting key statements or phrases and capturing the essence of the participant's experience as it relates to the phenomenon; formulating meaning by analyzing key statements to identify similar themes and patterns; developing themes by constructing themes from identified meanings; clustering similar themes together to create clusters and categories; creating a detailed description of the phenomenon; and returning results to participants to verify and check the correctness of interpretations (Morrow et al., 2015). In the present study, these steps were taken:

1. The transcript was read several times to gain a general sense. At this stage, all the thoughts, ideas, and assumptions about the hospital management during the COVID-19 pandemic were written in the bracketing file.
2. Significant phrases related to the vulnerability of the healthcare system were determined and extracted from each transcript, and then they were written and coded for analysis. Two researchers conducted a comparison of the extracted statements.
3. In this step, formulating meanings, the basic meanings were coded into a category to reflect a comprehensive description. Subsequently, the extracted meanings were compared with the original meanings in order to verify the consistency of the description by two authors.
4. When agreement about the formulated meanings was reached, the grouping of them was done. Each category contained interrelated meanings that reflected the structure of clusters of themes. Then, themes were formed by merging clusters of themes that were related to a common issue. At this stage, two researchers also checked the clusters of themes. An expert researcher in qualitative studies from another university (Bam University of Medical Sciences) controlled the data obtained at this stage.
5. All themes were comprehensively described. The essence of the phenomenon “the nurses’ lived experiences of vulnerability in hospital nursing management during the spread of COVID-19 pandemic” was extracted by incorporating all emerged themes. An expert researcher controlled the data obtained at this stage and confirmed the description of the themes.
6. At this stage, redundant or inappropriate descriptions that were vague and affected the overall description were modified or removed.
7. The final result is a comprehensive description of the phenomenon under study, confirmed by the participants (Morrow et al., 2015). The findings were sent through WhatsApp to the participants and necessary changes were made based on their opinions and suggestions.

Subsequently, by comparing and integrating the qualitative findings and the results of analyzing the summary reports of crisis management meetings, lessons and solutions, which were used by nursing managers during the COVID-19 pandemic, were obtained. The reports were reviewed by two authors independently, and if there was a disagreement, a third person was used. All extracted results were discussed in the research team. The researchers evaluated the data using the Consolidated Criteria for Reporting Qualitative Research.

### Rigor of the Study

To ensure the rigor of a study, Lincoln and Guba established a set of criteria for qualitative studies, encompassing credibility, dependability, confirmability, and transferability (Forero et al., 2018). To achieve the level of rigor in the present study, specific measures were implemented in accordance with Lincoln and Guba's evaluative criteria. Participants were selected with maximum variation to increase data transferability and credibility. Three transcribed interviews were returned to the participants to confirm the data extraction. Two external professional referees with doctoral degrees from Bam University of Medical Sciences performed an audition to validate the analysis process and to improve the dependability. Furthermore, in order to increase transferability, the authors focused on clarification and writing down a detailed description of the process of research methods and analysis (Babaei & Abolhasani, 2020; Faghani et al., 2023).

## Ethical Considerations

The Institutional Review Board and the Research Ethics Committee of the Iranshahr University of Medical Sciences approved the research proposal (IR.IRSHUMS.REC.1401.001). All necessary permissions were taken from the university hospital. Participants were informed of the objectives of the study and signed a written consent form before participation in the study. Participants choose the time, locations, and duration of the interview. The audio files were securely stored as encrypted files on the computer by the first author. Participation in the study was voluntary. Participants were assured that they could withdraw from the study at any time and that their information would be treated confidentially.

## Results

### Descriptive Results

Fourteen nurse mangers and staff nurses in different job positions participated in the study. The mean age of the participants was 38.5 ± 7.18 years. Participants’ characteristics are shown in Table 1.

**Table 1.** Characteristics of participants in the study (n = 14).

Participant number	Age (year)	Sex	Job position	Job experience (year)
1	39	Male	Matron (director of nursing services at hospital)	15
2	42	Male	Supervisor	18
3	46	Male	Supervisor	24
4	34	Male	Clinical supervisor	10
5	53	Male	Clinical supervisor	21
6	39	Female	Head nurse	13
7	41	Female	Head nurse	17
8	33	Female	Head nurse	10
9	44	Male	Head nurse	19
10	45	Male	Head nurse	22
11	28	Male	Nurse	6
12	29	Female	Nurse	7
13	32	Female	Nurse	8
14	34	Female	Nurse	11

### Qualitative Results

After the data was analyzed, 440 codes were extracted. After reducing, deleting, and merging the codes during the analysis process, 320 codes remained. Finally, 15 subthemes, four themes, and one overarching theme were extracted.

The results indicate that *threats to healthcare organization's management during the spread of COVID-19* was essence of the phenomenon. The essence is grounded in four themes: nurses’ attrition, distrust of society to the organization, the fragility in the organization's performance, and the intensified inequalities. Inadequate preparation for the global crisis, lack of planning, and the horrible shadow of death, disease, and anxiety caused multiple crises, including a shortage of healthcare personnel, evasion of responsibility, and a neglect of professional ethics and organizational standards. Furthermore, the spread of fake news and the public attention to local treatments posed additional threats to hospital management and public health.

#### Overarching Theme: Threats to Healthcare Organization's Management during the Spread of COVID-19

The overarching theme of the study was “Threats to healthcare organization's management during the spread of COVID-19.” The spread of COVID-19 was associated with several organizational risks for different reasons, including the highly contagious and unknown nature of the disease, the high morbidity and mortality, the unpredictability of the disease peaks, and lack of sufficient equipment and resources. Despite the daily efforts made at the hospital, many issues emerged that confused healthcare recipients, healthcare providers, and even nursing managers. In response to the question of the study: “How was the vulnerability in hospital nursing management during the spread of COVID-19?” four themes from the overarching theme of the study were extracted, consisting of “nurses attrition,” “distrust of society to the organization,” “fragility in the organization's performance,” and “intensified inequalities” (Table 2).

**Table 2.** Challenges of nurse managers and staff nurses in the University of Medical Sciences.

Overarching theme	Theme	Subtheme	Quotations
Threats to healthcare organization's management during the spread of COVID-19	Nurses attrition	Shadow of morbidity and mortality	“Some young nurses who just started working in the hospital came to the university nursing office with their family members and demanded they be allowed to quit their job. Even their family members said that if the university does not agree, we will not allow her to work as a nurse in the hospital and lose her life.” (Participant 1: Director of nursing services). “News of the deaths of nurses and medical staff in our hospitals, at home and around the world has become a great concern to hospital medical staff and nursing managers. Because of the severe shortage of nurses, their sickness or death and temporary or permanent leave had serious consequences for the hospital's condition. 25 nurses were infected and two departments of the hospital were on the verge of closing during one of the COVID-19 peaks.” (Participant 2: Supervisor)
		Migration magnetism	“One of my classmates moved to Germany and left the hospital. I am also collecting my documents to apply for immigration. Now is the best time. Once COVID-19 is over, it will not be as easy as it is now to be accepted in the destination country.” (Participant 12: Staff nurse)
		Overcoming emotions over responsibility	“Unfortunately, some nurses and their families were reluctant to continue their work due to the unknown and contagious nature of COVID-19, negative news from around the world, and the lack of a specific medication before developing the vaccines. Some nurses have come up with excuses for not working with COVID-19 patients.” (Participant 1: Director of nursing services)
	Distrust of society to the organization	Virulence of false news	“The spread of rumors and fear regarding hospitalization and mortality led to a devastating impact of the COVID-19 disease, resulting in a significant number of late admissions and subsequent fatalities.” (Participant 7: Head nurse)
		The superiority of cultural beliefs	“I can remember that during the fifth COVID-19 crisis, the number of patients increased significantly and hospital beds were all filled. In addition, the cultural beliefs of the region made people afraid to be hospitalized. They believed that their life and death were related to their destiny and the power of God. They also used different herbs for the treatment of COVID-19 in this area. Thus, we had to infuse medications for patients in an outpatient ward at the hospital so that they could leave the hospital after two hours. That was how people got treatment at our hospital.” (Participant4: Clinical supervisor)
		Whipping criticism of the healthcare organization's performance	“On the one hand, hard work in a crisis situation was difficult, and on the other hand, criticisms in virtual space destroyed the system. They ruthlessly criticized all of our actions. This unjustified criticism reduced our energy to continue.” (Participant 8: Head nurse)
	Fragility in the organization's performance	Instability in providing care and treatment	“One of the problems that we faced was changing treatment protocols. They might recommend a method to improve the patient's condition, we would teach it, then the protocol would be refused completely. We found ourselves in a confusing situation where treatment, medication, and related care were not stable.” (Participant 5: Clinical supervisor)
		Disorganized and emotional management	“Due to the unknown nature of the disease and the treatment, mortality, and stress, managers sometimes made entirely emotional decisions. These decisions have resulted in patient harm in some cases. As the patient's condition worsened, some managers became confused about whether to let the patient undergo imaging tests, and even made decisions that put the patient at higher risk.” (Participant 3: Supervisor)
		Moving away from organizational standards	“Due to the number of hospitalized patients and the need for intensive care, it was necessary to consolidate several medical and surgical departments and allocate them to the COVID-19 ward. The outpatient emergency room had to be converted to a COVID-19 ward, and the hospital delayed admitting some patients due to a lack of available beds.” (Participant 1: Director of nursing services)
		Stuck in unplanned situations	“The coronavirus is causing us all sorts of problems and shows that we are totally unprepared. We have learned nothing about dealing with crisis situations and have been forced to make decisions that are sometimes disconcerting.” (Participant 10: Head nurse)
		Developing crisis circles	“With a limited number of employees, it was difficult to plan. There is not enough work power for the outpatient department. No new staff were placed in the wards, just a few more anesthesiologists were added, which did not really solve the problem… Oxygen pressure dropped, and power outages caused problem. Supervisors disconnected all manometers from non-COVID wards and sent them to the ICU.” (Participant 4: Clinical supervisor)
		Diffusion of anxiety	“With the increasing number of hospitalized patients, the shortage of beds in the intensive care unit, and the sickness of the medical staff, I wondered when all the medical staff would die, and the hospital would fall into an unprecedented crisis.” (Participant 9: Head nurse)
	Intensified inequalities	Job-oriented inequalities	“Some of my colleagues were working shorter hours or working night shifts, and I felt that in the distribution of incentives, some staff were getting attention and others were not.” (Participant 4: Clinical supervisor)
		Disputable professional rights	“Despite all the hardships and suffering, the treatment team considered itself obliged to care and treat the patients, but some people caused more problems for the hospitals by not wearing masks and traveling, and it even reached a time when the hospital did not have the capacity to accept patients.” (Participant 7: Head nurse) Another problem that was experienced, was ignoring the efforts of nurses working in non-COVID-19 departments. “In this crisis, the efforts of nurses in non-COVID-19 wards were being ignored. If we hadn't worked with a limited number of staff in the hospital, the COVID-19 department would never have existed. We worked long, tight shifts with limited free time. Nurses in the COVID-19 ward were put on mandatory leave, but we weren't allowed to go on vacation.” (Participant 11: Nurse)
		Acceptance of moral conflicts	“Several anesthesiologists did not resuscitate some COVID-19 patients, believing their efforts were in vain. Gradually, with an increasing number of hospitalized patients, we have come to accept that COVID-19 patients will eventually die and should not be resuscitated, and that COVID-19 patients with severe lung disease and a poor prognosis do not require resuscitation.” (Participant 5: Clinical supervisor)
		Disruption in non-COVID-19 patients’ rights	“Because of department consolidation and bed shortages, patients were admitted to the department for only a short time and they tried to get the patient treated in the shortest possible time.” (Participant 6: Head nurse)

#### Theme 1: Nurses Attrition

This theme was classified into the three subthemes, including “shadow of morbidity and mortality,” “migration magnetism,” and “overcoming emotions over responsibility.”

##### 1.1. Shadow of Morbidity and Mortality

One of the biggest challenges associated with COVID-19 was morbidity and mortality among healthcare workers, especially nursing staff. The situation caused a great deal of stress across hospitals and exacerbated the problem of hospital nurse shortages and a disproportionate nurse-patient ratio during the COVID-19 crisis. Fear of death affected the lives of nurses and even the quality of patient care in hospitals.

##### 1.2. Migration Magnetism

Another risk of nurses’ attrition in the COVID-19 crisis was the wave of quitting their jobs in the hospital and migrating to other countries, which had an adverse effect on hospitals and healthcare systems. There were many reasons why nurses migrate, including: faster acquisition of residency permits in other countries, different working conditions and higher salaries, and no need for official language certification in the target countries.

##### 1.3. Overcoming Emotions Over Responsibility

Another issue that led to a decline in nursing staff during the COVID-19 time was that nurses feared caring for COVID-19 patients. As a result, some nurses left the nursing profession without even considering the mission of their profession.

#### Theme 2: Distrust of Society to the Organization

Several factors undermined the hospital's credibility during COVID-19. These factors were presented with three subthemes, including “virulence of false news,” “the superiority of cultural beliefs,” and “whipping criticism of the healthcare organization's performance.”

##### 2.1. Virulence of False News

During the COVID-19 pandemic, rumors gained momentum and affected people's minds. Some participants believed that COVID-19 patients in hospitals would receive inadequate medical care and die.

##### 2.2. The Superiority of Cultural Beliefs

The cultural beliefs of the people of this region caused the hospital crisis. Lack of belief in COVID-19 and the coronavirus, nonadherence to preventive procedures, avoidance of treatment, and use of only herbal and local remedies led to a significant increase in the number of cases.

##### 2.3. Whipping Criticism of the Healthcare Organization's Performance

Despite hospital-wide efforts to manage COVID-19 as efficiently as possible, those attempts were ineffective. Not enough resources, high patient mortality, and lack of specific infrastructure led to growing dissatisfaction on both sides, among people in the community and hospital as well as healthcare providers in the hospital. The hospital's performance was heavily criticized in virtual media. This criticism contributed to the poor performance of hospital managers and medical staff.

#### Theme 3: Fragility in the Organization's Performance

The stability of an organization during the COVID-19 pandemic was affected by many factors. These factors fall into five subthemes: “instability in providing care and treatment,” “disorganized and emotional management,” “moving away from organizational standards,” “stuck in unplanned situations,” “developing crisis circles,” and “diffusion of anxiety.”

##### 3.1. Instability in Providing Care and Treatment

The most challenging issue facing medical staff at this hospital and around the world was the lack of an effective treatment for the disease. This pandemic brought about rapid changes in protocols, trials and errors in medication and treatment, and trying to produce vaccines. These caused widespread confusion and frustration at all levels of management, treatment, and care in the healthcare organization.

##### 3.2. Disorganized and Emotional Management

Many factors influenced the operations of organizations during the COVID-19 crisis, such as a highly contagious and lethal disease, absence of comparable expertise and preparedness, insufficiency of equipment and infrastructure, scarcity of medical personnel, abrupt surge in the number of admitted patients, and the imbalance between hospitalized individuals and the available resources and workforce, fear and anxiety within the hospital and society. The confusion caused by the lack of an effective and coherent protocol, along with the resulting psychological burden, further exacerbated the situation. So, crisis management and decision making both were unplanned and highly emotional.

##### 3.3. Moving Away from Organizational Standards

The COVID-19 crisis occurred in an environment that even before the pandemic had some restrictions. This hospital was the only admission center for all cases, except for gynecology and pediatric. The number of hospital beds was limited in proportion to the covered population. With the COVID-19 pandemic, a number of departments were allocated to COVID-19 patients and many elective surgeries were canceled. The shortages of beds and ventilators, oxygen supplies, and power outages were intensified. Even the morgue did not match the number of patients who died due to the increased number of deaths caused by COVID-19 and other cases such as trauma and other cases.

##### 3.4. Developing Crisis Circles

The COVID-19 pandemic coincided with the emergence of multiple crises linked together like a chain. One of the main problems of the COVID-19 pandemic was the multiple outbreaks and different mutations with different strengths and virulence, posing great challenges for the management of the organization. Thus, the organization sometimes had to deal with simultaneous crises, such as the number of cases, deaths, and crises caused by multiple staff contracting COVID-19 at the same time. Additionally, insufficient infrastructure, electricity, oxygen, morgue capacity, medicines, and equipment were crises that managers faced during the COVID-19 pandemic.

##### 3.5. Diffusion of Anxiety

For a number of reasons, management during the COVID-19 pandemic was accompanied by anxiety and turmoil during the pandemic, which created a kind of pervasive uncertainty within the organization. The pandemic, people's failure to follow personal protection protocols, lack of definitive treatment, changes in new treatment protocols and previous protocols, staff sickness and death of staff, inadequate infrastructure, and lack of predictive planning led to future uncertainty and deep anxiety on all levels of management and care.

#### Theme 4: Intensified Inequalities

This theme discussed conflicts that resulted in inequalities regarding the rights of medical staff and patients within the organization. It includes four subthemes: “job-oriented inequalities,” “disputable professional rights,” “acceptance of moral conflicts,” and “disruption in the non-covid-19 patients’ rights.”

##### 4.1. Job-Oriented Inequalities

The COVID-19 pandemic was associated with several rights violations and disputes. It was a difficult decision to select medical staff to work in the COVID-19 wards. Some staff did not want to work there, but the needs of patients forced managers to make decisions, sometimes placing staff in COVID wards against their will. Also, there were occasional instances of unfairness. Shift planning, the allocation of staff incentives, and giving all staff fair leave were challenges.

##### 4.2. Disputable Professional Rights

Under the influence of cultural factors, distrust of treatment, the illusion of not getting sick, and treatment with herbal medicines, all contributed to nonadherence to personal hygiene protocols. This noncompliance led to an increase in the number of cases and deaths, increased the burden on hospitals, and caused infections and deaths among medical staff.

##### 4.3. Acceptance of Moral Conflicts

For several reasons, medical staff faced conflicts and moral distress during the COVID-19 pandemic. Lack of resources, allocation of available resources according to patient prognosis and prioritization, not performing cardiopulmonary resuscitation of some patients, and fear and avoidance of caring for patients are instances that happened during the COVID-19 pandemic.

##### 4.4. Disruption in the Non-COVID Patients’ Right

The high number of COVID-19 patients, limited resources, ward consolidation, and a shortage of medical staff as well as focusing on COVID-19 patients adversely affected the rights of non-COVID-19 patients.

## Four Crisis Management Lessons

### 1. Brain Drain Was Intensified among Nurses and Other Healthcare Personnel

*Solution:* Some measures to prevent immigration of nurses included standardizing the number of workforce in each department, balancing the working hours, reducing working overtime, and employing graduate specialized nurses in the departments as well as the extension of 1-year governmental contract of nurses to work in the hospital.

### 2. The Cultural Challenges of the Society and the Power of Rumors, Caused Distrust of the Society to the Hospital

*Solution:* Nurse managers considered the dominant culture of the region. They sent several messages by regional media and made changes to patients’ admissions. They opened five outpatient clinics outside of the main building of the hospital and patients were visited by the physicians and nurses in the clinics to deliver appropriate antiviral medicines to them, which led to a significant increase in outpatient admissions and a decrease in patients’ deaths. Also, in the outpatient clinics, an educational pamphlet was delivered to the patients and their families to explain the advantages of the classical treatments and disadvantages of traditional medicines for COVID-19 treatment.

### 3. Nurse Managers Experienced Fragility in the Organization Management

*Solution:* Nurse managers were involved in the university strategic planning meetings during the COVID-19 pandemic and after that. Also, the management style of the nurse managers underwent changes at the national and hospital levels. Nurse managers at the hospital established regular skyroom meetings with other counterparts and shared their decisions and positive actions or ineffective interventions throughout the country.

### 4. Inequalities Were Increased Within the Healthcare Organization

*Solution:* Volunteer specialist healthcare providers were recruited in the hospital. Also, the inpatient ward for COVID-19 patients was reopened, and two COVID-19 Intensive Care Units were established during this period.

## Discussion

The present study aimed to explore the lived experiences of nurse managers and staff nurses regarding vulnerability in hospital nursing management during the COVID-19 pandemic and reflect on crisis management lessons in our society. This qualitative study showed the nurse managers’ leadership challenges and important insights for addressing the challenges during the COVID-19 crisis. These findings can serve as a foundation for organizational preparedness and implementing agendas in comparable situations both presently and moving forward.

The results of our study revealed that the COVID-19 pandemic was accompanied by numerous challenges for nurse managers which were a threat to the healthcare system. This issue happened around the world. In a cross-sectional study, 96.7% nurse managers reported that COVID-19 has brought new challenges for them (Gab Allah, 2021). The COVID-19 crisis was described as a constant concern for contemplation by nurse managers in Finland (Ahlqvist et al., 2023). One of the challenges highlighted in the present study was the negative impact of the COVID-19 pandemic on the number of nurses, which further exacerbated the previous shortage of nurses. In previous qualitative studies in Egypt, Canada, Spain, and Finland, nursing managers reported similar findings regarding the impact of the COVID-19 pandemic on the shortage of nursing staff (Ahlqvist et al., 2023; Hossny et al., 2022; Udod et al., 2024). Absenteeism, fear of infection, anxiety, and infection among nurses as well as work-overload and extra work, were described as challenges and contributing factors to the worsening shortage of nurses in both qualitative and quantitative studies (Gab Allah, 2021; Hossny et al., 2022; Vázquez-Calatayud et al., 2022). In Hossny et al.'s study in Egypt, nurse managers explained the difficulty of traveling to the workplace due to quarantine and travel laws as a reason for absenteeism during COVID-19 (Hossny et al., 2022). In addition to these factors, studies have mentioned various factors that have an impact on the reduction of the number of nurses during the COVID-19 pandemic, including the complex nature of the disease and its complications, exposure to and work with suspected and positive cases, psychological problems, caring refusal due to fear of being infected and spreading it to their families, as well as job turnover during COVID-19, burnout, and inadequate opportunities and wages (Hossny et al., 2022; Kazemi et al., 2023; Quadros et al., 2021). Also, Egyptian nurse managers mentioned the protection of their children and family members as a source of psychological problems during the COVID-19 pandemic (Hossny et al., 2022). Evidence shows that personal and family members’ health has been one of the concerns of healthcare providers, especially when the family members are elderly or ill (Ardebili et al., 2021). Nurse managers in Finland and Spain explained that managing staff, ensuring the existence of a sufficient number of personnel, and providing instruction led to the intensification of the managers’ workload. Organizing staff for each working shift when some of the personnel were sick, had positive test for COVID-19, or were on leave, would lead to changes in the daily program and challenges for nurse managers (Ahlqvist et al., 2023; Vázquez-Calatayud et al., 2022).

On top of that, payment of higher salaries and bonuses in some healthcare organizations, caused nurses to leave their jobs. This led managers to apply for additional payment incentives in some positions that were very difficult to fill (Pahlevan Sharif et al., 2023). Migration was another reason for nurses’ attrition. COVID-19 increased the demand for nurses globally, and many countries accepted immigrant nurses (Shaffer et al., 2022). Healthcare providers migrated from developing countries to developed ones over the years. But, after the COVID-19 pandemic, almost a quarter of healthcare professionals reported their increased intention to migrate. Improved quality of life, increased income and access to social and professional opportunities were among the factors influencing the migration motivations of healthcare providers (Murataj et al., 2022).

Distrust of society in the healthcare organization was another important threat for organizational management in our context. Based on our participants’ experiences, not enough preparedness and organized planning for COVID-19 pandemic not only damaged our hospital's reputation and performance, but also irreparably damaged our human resources. Thus, confidence in our hospital health recovery was undermined by inconsistent care and poor outcomes. COVID-19 wreaked havoc on societies and healthcare organizations, and dealing with uncertainty, was one of the greatest challenges during the COVID-19 crisis (Wu et al., 2020). Uncertainty in the COVID −19 pandemic was reported by nurse managers in different studies (Ahlqvist et al., 2023; Gab Allah, 2021; Udod et al., 2024). Ambiguity and uncertainty as current challenges were reported during the COVID-19 crisis by 90.7% of nurse managers (Gab Allah, 2021). In addition, the top challenges were reported the image of society and difficult decisions by nurse managers, and also a smaller percentage of them mentioned rumors as a challenge for managers (Gab Allah, 2021). In a qualitative study, Finish nursing managers attributed their uncertainty to lack of understanding, unpredictability, and ongoing uncertainty about the COVID-19 pandemic. In addition, quick decisions and fast actions in a short time frame were added to the challenges of them (Ahlqvist et al., 2023). These factors were detrimental to and undermined the performance of nursing organizations, and left them vulnerable

According to our results, various ethical conflicts arose during the pandemic in our context, manifesting as disregard for caregiver and patient rights. The right of patients to receive quality care and even the right of nurses to maintain their own health were undermined. This concern created moral distress and ongoing pressure for nurses. In addition, inequitable distribution of scarce resources and tense relationships between nurses and patients and their families were also sources of distress (Gebreheat & Teame, 2021). In Udod's study, moral burden was an issue for both staff and managers during the COVID-19 pandemic. A moral burden for the participants was due to facing issues such as the safety of patients, staff, and the staff's families. Different factors such as uncertainty, longer work hours, and additional responsibilities, as well as experiencing patient suffering and death, led to nursing staff being more anxious, exhausted, and demoralized (Udod et al., 2024). In addition, nurse managers in Finland reported addressing insecurities, limiting extra work tasks, and receiving feedback and critique during the chaotic nature of the COVID-19 crisis (Ahlqvist et al., 2023). Sperling, in his study, showed that the majority of nurses complained of inadequate support and protection in their workplace (Sperling, 2021). Finish nursing managers also described it as difficult to take care of personnel needs during the COVID-19 pandemic (Ahlqvist et al., 2023). High workload, uncertainty and caregiving in difficult situations, compromised therapeutic relationships between nurses and patients, nurses’ avoidance of caring, and inequalities at work were also reported as ethical conflicts (Heydarikhayat et al., 2022; Sperling, 2021).

## Strengths and Limitations of the Study

The present study has several strengths. First, this study was carried out during the seven wave of the pandemic, which may have led to reaching deep perceptions of individual perspectives regarding the process of facing the COVID-19 pandemic from the beginning, to passing through different stages and learning from national and global experiences in providing the best possible care in a context with many limitations. In addition, understanding the experiences of nurse managers at different levels can help identify the challenges and opportunities as a basis for better planning in the future. Third, we interviewed managers and took into consideration staff nurses at the front line of care to hear their experiences and to deepen the extracted findings. Regarding limitations, this study was conducted within a single healthcare organization, thus, the results may not be transferable to another healthcare settings. We focused on only the experiences of nursing managers at the university hospital and did not use the experiences of university managers.

## Future Research Recommendations

The importance of culture of the context and professional ethics should also be emphasized. National and international collaboration is necessary in planning for crises. More studies are required on how to manage the challenges and vulnerabilities of the healthcare organizations with multicircle limitations during the pandemic.

## Implications for Practice

The findings of this qualitative study indicate that clear and well-organized crisis management plans can support the leadership process of nursing managers in future crises. Also, they can help preserve the assets and credibility of the healthcare system. The migration of nurses and brain drain in the COVID-19 pandemic and the continuation of migration after the crisis are a warning for nursing managers to reanalyze the current situation of nursing personnel. In the context of a pandemic, behaviors such as the evasion of responsibility have appeared among nurses for different reasons. It is imperative that nursing managers prioritize future plans with the aim of restructuring programs that emphasize professional ethics of nursing and acceptance and internalization by nurses.

## Conclusions

This study provides a detailed description of nurse managers and staff nurses experiences regarding the vulnerability in hospital nursing management during the COVID-19 pandemic. A university hospital during a pandemic was under a considerably complex situation for leadership work. The unpreparedness of healthcare system for a crisis led to the loss of organizational assets, including medical staff and the credibility of the healthcare system. The mistrust of service recipients resulted in the person's tendency for nonstandard treatments and the temporary dominance of cultural beliefs over local treatments and irreparable damage. Like the pressure that deepens the cracks on a glass, the COVID-19 pandemic made the vulnerabilities of a healthcare system more obvious. Weaknesses and limitations of the infrastructure became more obvious during the pandemic and became serious threats to the healthcare system. The dissatisfaction of nurses was revealed by migration and trying to find better facilities and better working conditions and their preferences over others by escaping from care and not internalizing ethical issues in patient care. Despite severe challenges along with the pandemic, it offered valuable nursing management lessons. These lessons can probably contribute to better planning by healthcare managers in our society and other parts of the world to prevent weakness in the organizational performance during crisis development. Self-awareness and insight into the weaknesses of the healthcare organization and the participation of nursing managers along with senior university managers helped them to find practical solutions to solve serious issues in the crisis and provided impressive lessons for comprehensive crisis management and health promotion in hospitals.

## Supplemental Material

sj-docx-1-son-10.1177_23779608241286308 - Supplemental material for Exploring Lived Experiences of Vulnerability in Nursing Management during the Coronavirus Disease 2019 Pandemic: A Phenomenological Study of Nurse Managers and Nurses Supplemental material, sj-docx-1-son-10.1177_23779608241286308 for Exploring Lived Experiences of Vulnerability in Nursing Management during the Coronavirus Disease 2019 Pandemic: A Phenomenological Study of Nurse Managers and Nurses by Nastaran Heydarikhayat, Nezar Ghanbarzehi, Fatemeh Darban, Zahra Atarodi Kashani and Camelia Rohani in SAGE Open Nursing

sj-docx-2-son-10.1177_23779608241286308 - Supplemental material for Exploring Lived Experiences of Vulnerability in Nursing Management during the Coronavirus Disease 2019 Pandemic: A Phenomenological Study of Nurse Managers and Nurses Supplemental material, sj-docx-2-son-10.1177_23779608241286308 for Exploring Lived Experiences of Vulnerability in Nursing Management during the Coronavirus Disease 2019 Pandemic: A Phenomenological Study of Nurse Managers and Nurses by Nastaran Heydarikhayat, Nezar Ghanbarzehi, Fatemeh Darban, Zahra Atarodi Kashani and Camelia Rohani in SAGE Open Nursing
