# Supplementary material for: Exploring Lived Experiences of Vulnerability in Nursing Management during the Coronavirus Disease 2019 Pandemic: A Phenomenological Study of Nurse Managers and Nurses
Source: SAGE Open Nurs. 2024 Oct 8;10:23779608241286308. doi: 10.1177/23779608241286308 (PMC11462562; doi:10.1177/23779608241286308)
Supplement: sj-docx-1-son-10.1177_23779608241286308 - Supplemental material for Exploring Lived Experiences of Vulnerability in Nursing Management during the Coronavirus Disease 2019 Pandemic: A Phenomenological Study of Nurse Managers and Nurses [file sj-docx-1-son-10.1177_23779608241286308.docx]

**Interview Guide**

**Introduction**

The COVID-19 pandemic placed enormous pressure on healthcare systems around the world, but this challenging period, has also provided valuable insights for healthcare organisations. The pandemic weakened even the strongest healthcare systems around the world, leading to a failure to properly manage and respond to the crisis.

**The objective of the study**

This study aimed to explore nurses’ lived experiences of vulnerability in hospital nursing management during the COVID-19 pandemic, and reflect on the lessons learned.

**Preparation for the Interview**

1. An appropriate setting is chosen for the interview process.

2. The objectives of the interview are clearly conveyed to the participant.

3. Consent, both verbal and written, is obtained from the participant for the purpose of recording the interview.

4. To ensure confidentiality, the participant is informed of their right to withdraw at any point

5. The participant is informed about the characteristics of the interview, including its structure and anticipated length.

6. Information is provided to the participant regarding how to contact the researcher for any subsequent questions.

7. The participant is encouraged to ask any questions prior to the commencement of the official interview.

**Pilot testing**

1. The interview starts with a warm welcome, an introduction, and appreciation for the participant's engagement in the process.

2. Open-ended questions are utilized throughout the interview.

3. The interview begins with general inquiries, and as responses are provided, it transitions into more detailed exploration.

4. The use of leading questions is deliberately avoided.

5. The interview is facilitated through follow-up and exploratory strategies, including techniques like Silent Probe and Echo Probe.

6-Questions that start with "why" are raised with caution because these types of questions end up with a cause and effect relationship that may not be valid.

Starting the interview and directing

7- During the interview, the interviewer acts as neutral as possible and avoids displaying strong emotional reactions to the participant's answers.

8- Only one question is asked at a time to maintain focus.

9- Inquiries regarding the present tense are prioritized over those concerning the past and future.

10- Between the important topics, a transition period is suggested: like now, I would like to address this issue..."

**Areas of interview**

1. The experiences of nurse managers regarding their sense of vulnerability in the context of hospital nursing management during the spread of COVID-19 pandemic.

2. The experiences of staff nurses related to vulnerability in nursing management during the spread of COVID-19 pandemic.

3. The challenges encountered by nurse managers in their professional management role throughout the COVID-19 pandemic.

4- Factors aggravated the challenges of nurses’ management during the spread of COVID-19 pandemic

**Sample questions**

| Main questions |
| --- |
| 1. Please explain how did you experience vulnerability in nursing management during the spread of COVID-19 pandemic?” |
| 1. As a nurse manager, what challenges did you experience in your professional management during the spread of COVID-19? |
| 1. As a staff nurse, what challenges did you experience in the ward or hospital due to nursing management during the spread of COVID-19? |
| 1. What was your limitations in your professional work? |
| 1. How did you experience access to the resources in the hospital during the spread of COVID-19? |
| 1. What have been the effects of vulnerability on the hospital and community? |
| 1. What factors aggravated the challenges of nursing management during the spread of COVID-19 pandemic? |
| 1. Based on your experience, which factors had exacerbated vulnerability in hospital nursing management? |
| Probing |
| 1. Can you explain more? |
| 1. What did you mean in this case? |
| 1. Could you please give me an example? |
| 1. Is there anything else you want to say? |

**End of the interview**

1. The participant is inquired whether there are any topics not covered during the interview that he/she wishes to discuss further.

2. If she/he has a special suggestion in this field, the researcher will be eager to know about them.

3. We extend our heartfelt thanks to the participant for his generous allocation of time.

**Reference**

Turner, D. W. (2010). Qualitative Interview Design: A Practical Guide for Novice Investigators. The Qualitative Report, 15(3), 754-760. <https://doi.org/10.46743/2160-3715/2010.1178>
